# Supplementary material for: Nanoscale synchrotron x-ray analysis of intranuclear iron in melanised neurons of Parkinson’s substantia nigra
Source: Commun Biol. 2024 Aug 20;7:1024. doi: 10.1038/s42003-024-06636-1 (PMC11335769; doi:10.1038/s42003-024-06636-1)
Supplement: Supplementary file 2 — Reporting summary [file 42003_2024_6636_MOESM2_ESM.pdf]

nature portfolio

Corresponding author(s): Dr Jake Brooks

Last updated by author(s): Jun 5, 2024

Reporting Summary

Nature Portfolio wishes to improve the reproducibility of the work that we publish. This form provides structure for consistency and transparency in reporting. For further information on Nature Portfolio policies, see our Editorial Policies and the Editorial Policy Checklist.

Statistics

For all statistical analyses, confirm that the following items are present in the figure legend, table legend, main text, or Methods section.

n/a	Confirmed	
☒	☐	The exact sample size (n) for each experimental group/condition, given as a discrete number and unit of measurement
☒	☐	A statement on whether measurements were taken from distinct samples or whether the same sample was measured repeatedly
☒	☐	The statistical test(s) used AND whether they are one- or two-sided<br>*Only common tests should be described solely by name; describe more complex techniques in the Methods section.*
☒	☐	A description of all covariates tested
☒	☐	A description of any assumptions or corrections, such as tests of normality and adjustment for multiple comparisons
☒	☐	A full description of the statistical parameters including central tendency (e.g. means) or other basic estimates (e.g. regression coefficient) AND variation (e.g. standard deviation) or associated estimates of uncertainty (e.g. confidence intervals)
☒	☐	For null hypothesis testing, the test statistic (e.g. F, t, r) with confidence intervals, effect sizes, degrees of freedom and P value noted<br>*Give P values as exact values whenever suitable.*
☒	☐	For Bayesian analysis, information on the choice of priors and Markov chain Monte Carlo settings
☒	☐	For hierarchical and complex designs, identification of the appropriate level for tests and full reporting of outcomes
☒	☐	Estimates of effect sizes (e.g. Cohen's d, Pearson's r), indicating how they were calculated

Our web collection on statistics for biologists contains articles on many of the points above.

Software and code

Policy information about availability of computer code

Data collection	Data was collected at Diamond Light Source synchrotron facility using Generic Data Acquisition (GDA) software. GDA provides a common Python syntax scripting environment with Graphics User Interface and Object customisations, used for data acquisition at most Diamond Light Source beamlines.
Data analysis	aXis 2000 software package, Oct2019 version (available at http:/unicorn.mcmaster.ca/aXis2000.html) was used for processing and analysis of x-ray spectromicroscopy data. Java-based image processing program, ImageJ version 2.1.0/1.53c, was used to apply a bandpass filter to images and to create pseudo-coloured composite images.

For manuscripts utilizing custom algorithms or software that are central to the research but not yet described in published literature, software must be made available to editors and reviewers. We strongly encourage code deposition in a community repository (e.g. GitHub). See the Nature Portfolio guidelines for submitting code & software for further information.

Data

Policy information about availability of data

All manuscripts must include a data availability statement. This statement should provide the following information, where applicable:

- Accession codes, unique identifiers, or web links for publicly available datasets
- A description of any restrictions on data availability
- For clinical datasets or third party data, please ensure that the statement adheres to our policy

The data that support the findings in this study will be available in the Warwick Research Archive Portal (WRAP) repository at https://wrap.warwick.ac.uk/185993

Research involving human participants, their data, or biological material

Policy information about studies with human participants or human data. See also policy information about sex, gender (identity/presentation), and sexual orientation and race, ethnicity and racism.

Reporting on sex and gender	Reporting on sex and/or gender was not relevant as comparing groups was not a central aim of this study (see study design section below).
Reporting on race, ethnicity, or other socially relevant groupings	Reporting on race and/or ethnicity was not relevant as comparing groups was not a central aim of this study (see study design section below).
Population characteristics	Reporting on population characteristics was not relevant to this study due to the small sample size (see study design section below).
Recruitment	No living research participants were involved in this study. Brain tissue material was selected based on diagnostic neuropathology performed at the tissue bank (Newcastle Brain Tissue Resource).
Ethics oversight	Human brain tissue used in this study was analysed in accordance with the Declaration of Helsinki under the remit of ethical approvals REGO-2018-2223 from the BSREC at University of Warwick, and approval 07/MRE08/12 from the North West Haydock Ethics Committee

Note that full information on the approval of the study protocol must also be provided in the manuscript.

Field-specific reporting

Please select the one below that is the best fit for your research. If you are not sure, read the appropriate sections before making your selection.

☒ Life sciences ☐ Behavioural & social sciences ☐ Ecological, evolutionary & environmental sciences

For a reference copy of the document with all sections, see nature.com/documents/nr-reporting-summary-flat.pdf

Life sciences study design

All studies must disclose on these points even when the disclosure is negative.

Sample size	The work presented constitutes an exploratory, proof-of-concept, study to evaluate the utility of synchrotron x-ray spectromicroscopy for intranuclear iron characterisation. As such, a single case (n=1) was used to demonstrate this.
Data exclusions	No data exclusions
Replication	Intranuclear iron was consistently shown to be in the same ferric oxidation state across multiple neurons measured. Repeat scans were also used to verify scanning parameters, with no evidence of beam-induced changes to the iron oxidation state. This confirmed measurements to be reproducible.
Randomization	Randomisation was not relevant to this study due to the small sample size
Blinding	Blinding was not relevant to this study due to the small sample size

Reporting for specific materials, systems and methods

We require information from authors about some types of materials, experimental systems and methods used in many studies. Here, indicate whether each material, system or method listed is relevant to your study. If you are not sure if a list item applies to your research, read the appropriate section before selecting a response.

Materials & experimental systems

n/a	Involved in the study
☒	☐ Antibodies
☒	☐ Eukaryotic cell lines
☒	☐ Palaeontology and archaeology
☒	☐ Animals and other organisms
☒	☐ Clinical data
☒	☐ Dual use research of concern
☒	☐ Plants

Methods

n/a	Involved in the study
☒	☐ ChIP-seq
☒	☐ Flow cytometry
☒	☐ MRI-based neuroimaging

Plants

Seed stocks	*Report on the source of all seed stocks or other plant material used. If applicable, state the seed stock centre and catalogue number. If plant specimens were collected from the field, describe the collection location, date and sampling procedures.*
Novel plant genotypes	*Describe the methods by which all novel plant genotypes were produced. This includes those generated by transgenic approaches, gene editing, chemical/radiation-based mutagenesis and hybridization. For transgenic lines, describe the transformation method, the number of independent lines analyzed and the generation upon which experiments were performed. For gene-edited lines, describe the editor used, the endogenous sequence targeted for editing, the targeting guide RNA sequence (if applicable) and how the editor was applied.*
Authentication	*Describe any authentication procedures for each seed stock used or novel genotype generated. Describe any experiments used to assess the effect of a mutation and, where applicable, how potential secondary effects (e.g. second site T-DNA insertions, mosiacism, off-target gene editing) were examined.*
